# Structural Characterization and Peroxidation Stability of Palm Oil-Based Oleogel Made with Different Concentrations of Carnauba Wax and Processed with Ultrasonication

**DOI:** 10.3390/gels8120763

**Published:** 2022-11-23

**Authors:** Paramee Noonim, Bharathipriya Rajasekaran, Karthikeyan Venkatachalam

**Affiliations:** 1 Faculty of Innovative Agriculture and Fishery Establishment Project, Prince of Songkla University, Surat Thani Campus, Makham Tia, Mueang, Surat Thani 84000, Thailand; 2 International Center of Excellence in Seafood Science and Innovation, Faculty of Agro-Industry, Prince of Songkla University, Hat Yai, Songkhla 90110, Thailand

**Keywords:** palm oil, carnauba wax, ultrasonication, homogenization, structural characteristics, thermal properties, peroxidation, storage

## Abstract

The effect of ultrasonication (25 kHz for 10 min) on physical, thermal, and structural properties and storage stability of palm oil-based oleogels prepared using different concentrations of carnauba wax (CW) (5% or 10%) were investigated and compared with oleogels prepared with a homogenizer (2000 rpm for 10 min). Overall, this study found that applying an ultrasonication process with higher CW concentration (10%) effectively improved the properties and stability of palm oil-based oleogel (*p* < 0.05). Oleogels processed with ultrasonication had higher lightness (L*), higher yellowness (b*), and lower redness (a*) than those processed with homogenizer (*p* < 0.05), irrespective of CW concentrations. However, a higher CW concentration (10%) increased the textural properties of oleogels such as hardness, stickiness, and tackiness as compared to oleogels with a lower CW concentration (5%) (*p* < 0.05). Thermal properties including melting onset temperature, melting peak temperature, and melting enthalpy were found to be significantly higher in ultrasonication-processed oleogels with high CW concentration (*p* < 0.05). Furthermore, the microscopic examination of the oleogels exhibited a strong gel network when prepared using a high concentration of CW and processed with ultrasonication. Fourier Transform Infrared (FTIR) spectra of oleogels revealed that strong intra- and intermolecular interactions were formed by hydrogen bonding between CW and palm oil. X-ray diffraction (XRD) showed a smooth and fine structural network of oleogels and proved that ultrasonication increased the structural properties of oleogel. Moreover, oil loss and peroxide value of oleogels were increased during 90 days of storage (*p* < 0.05). However, oleogels processed with the ultrasonication had reduced oil loss and increased peroxidation stability during storage (*p* < 0.05). Overall, this study showed that application of ultrasonication with a higher CW concentration could improve properties and storage stability of palm oil-based oleogel.

## 1. Introduction

The physical state of edible fats and oils determines their physical forms, which range from a viscous liquid to a solid, hard fat. Food production generally relies on the physical state of these fats. As a result, fats and oils are typically integral to obtaining a variety of desired functionalities such as flavor, nutritional properties, food stability, tenderness, palatability, texture, color, food appeal, solubility, etc. [1]. Fats such as saturated fats and trans fats are responsible for most food functionalities. By stacking crystalline lamellae, a solid fat network is formed, resulting in crystalline nanoplatelets and entrapping liquid fat within the solid fat network [2]. Fats can function in this way because of the triacylglycerols in their structure. Despite providing numerous significant functions, it may cause consumers to experience several adverse health effects, particularly chronic diseases, and cardiovascular diseases are the most prevalent of these disorders [3]. The amount of natural solid fats, on the other hand, is relatively low. Margarine has been available in commercial markets for many decades, yet it can also cause adverse health effects, and consumers are aware of the chemical processes involved in its production [4]. Recently, several countries have implemented strict rules against using margarine or processed trans fatty acids to protect public health. Regarding trans fats, the United States Food and Drug Administration (FDA) concluded in 2015 that partially hydrogenated oils are not generally recognized as safe (GRAS) [5,6]. Interesterification is one of the alternatives to the synthesized solid fats production processes; however, all alternatives require chemical processing, chilling, and other polymorphic crystallization processes [7]. Edible oleogels have gained popularity in recent years due to their ease of preparation and superior health benefits over synthetic and natural saturated trans fats. Unlike solid fats, edible oleogels have elastic properties and are primarily composed of unsaturated fats, which are healthier and lower in less-healthy saturated fats. The majority of oleogels can be transformed from solution to gel numerous times regardless of the chemical nature of the oil, and the process can be achieved by simply reheating and recooling [8]. An oleogel forms fibrillar or platelet crystals by self-assembling in polar solvents through noncovalent interactions. It is also important to note that Van der Waals and electrostatic interactions contribute to the gelation of edible oils in addition to hydrogen bonds and stacking interactions. Oleogels are generally prepared by adding gelators to edible oil and then treating them with thermal processing [9]. However, recent research has found that ultrasonication can improve the gelling property of oleogels. Additionally, the application of ultrasonication could also help improve the microparticles’ dispersion and nanowire properties in oleogel [10]. Plant wax esters are among the most-used gelators in producing oleogels. These include beeswax, carnauba wax, candelilla wax, sunflower wax, and rice bran wax [11]. Even though oleogels are receiving considerable attention from researchers and producers, they have not made significant progress due to the limited availability of edible gelators and the high costs associated with large-scale production [12]. Carnauba wax, one of the edible wax gelators, is abundantly available and inexpensive, making it an attractive choice for producing oleogels. A major characteristic of carnauba wax is its ability to stabilize and crystallize. There has been extensive research on the efficacy and efficiency of carnauba wax as a gelator on various edible plant oils [13]. The extensive use of palm oil is noted as an inexpensive frying medium for a variety of foods around the world, and especially, their usage is more abundantly found in Asia, particularly in Southeast Asia, which is among the largest producers of palm oil. Although palm oil is abundant, very little information is available regarding the production and characterization of oleogel using palm oil. Our previous study examined the ability and stability of palm oil-based oleogel as a frying medium for instant noodles [10]. The results were very encouraging and indicated that the oleogel was safe for food use. Furthermore, unlike regular palm oil as a frying medium with a shorter shelf life, palm oil oleogel can be reused numerous times without increasing the high peroxidation rate and thermal degradations. However, structural characterization and thermal stability of palm oil-based oleogels prepared with carnauba wax are still lacking. In this study, solid-like oleogels were prepared using palm oil structured by carnauba wax at different concentrations (5% or 10%) and processed with ultrasonication. The effects of the ultrasonication on physical, thermal, and structural properties of palm oil-based oleogels were investigated and compared with those processed with a homogenizer. Moreover, the stability of oleogels was also evaluated during storage of 90 days at ambient temperature. This study provides a new idea for the preparation of palm oil-based oleogels with improved properties using ultrasonication.

## 2. Results and Discussion

### 2.1. Color and Textural Profiles

**Figure 1:**
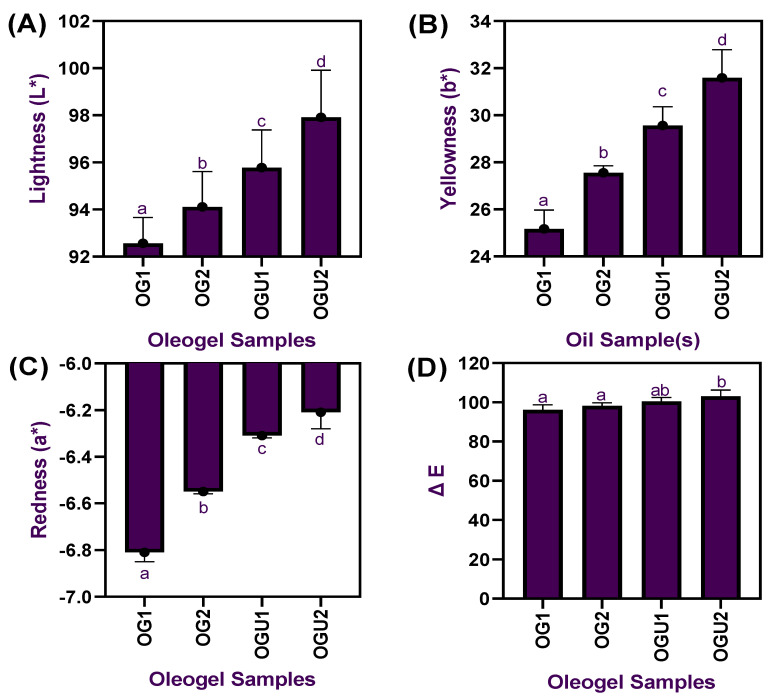
Lightness (**A**), yellowness (**B**), redness (**C**), and total color (ΔE) (**D**) of palm oil-based oleogels prepared with different concentrations of carnauba wax and the ultrasonication process.

**Figure 2:**
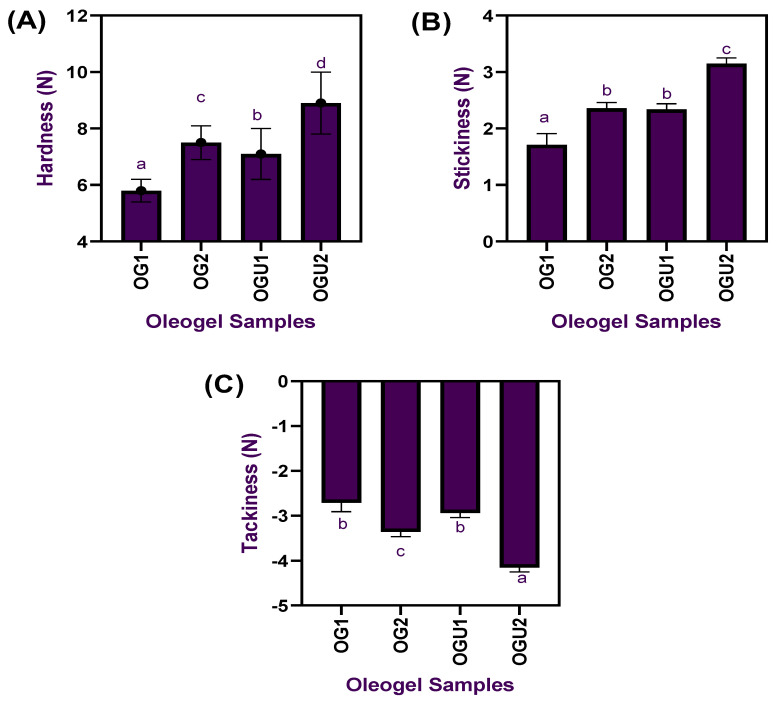
Hardness (**A**), stickiness (**B**), and tackiness (**C**) of palm oil-based oleogels prepared with different concentrations of carnauba wax and the ultrasonication process.

The color characteristics of the palm oil-based oleogel samples prepared with different concentrations of carnauba wax and the ultrasonication process are shown in Figure 1. Overall, the results exhibited significant differences in the tested color characteristics on the oleogels, regardless of the different concentrations of CW and ultrasonication process used (*p* < 0.05). The lightness of the oleogels significantly differed among each other, and, especially when the CW concentration increased to a high level, the lightness of the oleogels tended to be vastly increased (*p* < 0.05); in addition, the ultrasonication process also helped the oleogels in improving the overall lightness (*p* < 0.05). Similarly, the yellowness of the oleogels also tends to increase upon the increased concentration of CW (*p* < 0.05), and the ultrasonication process had further improved the yellowness in the oleogels, regardless of the wax concentrations used (*p* < 0.05). On the other hand, the redness values in the oleogels were in the opposite trend (*p* < 0.05). Generally, the color values in the oleogels mainly depend on the raw materials used, as it directly influences the oleogels’ color values. In our study, the palm oil was originally a light brownish yellow; however, when CW was used as a gelator in the oleogels emulsion, the color of the oleogel was turned into a brighter yellowish, and the addition of ultrasonication could have increased the microbubbles in the oleogel emulsion and helped to reflect more light as compared with the oleogels that are prepared traditionally without sonication. Chen et al. [14] reported that the ultrasonication process induces a strong scattering efficiency, reflecting more light, that is achieved by influencing the droplet size in the oleogels. Ögütcü et al. [15] reported that the total color values of the oleogels increased when the gelator concentration increased, and their study found that increased opaqueness of the oleogels played a crucial role in the increased total color values. This is in accordance with the present study, in which the increased concentration of CW could have increased the opaqueness values of the oleogel. Applying the ultrasonication process also increased the total color values of the oleogels. Pucas et al. [16] found that color values of the oleogels were not significantly improved when using candelilla wax as a gelator, and their study recommended using colorants for better attraction. It is indicated that not all the gelators could improve the color of the oleogels; however, in this study, the use of CW significantly improved the color profile of the oleogels, which is solely recommended for yellow color-based food applications. The textural properties of palm oil-based oleogel samples prepared with different concentrations of carnauba wax and the ultrasonication process are shown in Figure 2. Normally, the hardness of a wax oleogel is one of the main factors in determining the potential applicability of the wax oleogels. The hardness of the oleogel samples tended to increase when the oleogel emulsions were prepared with the ultrasonication process and higher CW concentrations as compared to the other tested samples in this study (*p* < 0.05). It indicates that the application of sonication and higher concentration of CW had significantly (*p* < 0.05) increased the oleogel strength and maintained a better oleogel network structure. Stickiness values in the oleogels represent the internal bonding strength and are also used to identify the resistance against external damage. This study showed that increased CW concentration significantly increased the oleogels’ stickiness values (*p* < 0.05). Additionally, similar effects were also found in the ultrasonicated oleogel samples (*p* < 0.05). Furthermore, the oleogels were also tested for tackiness properties. It was found that increased wax concentration and application of sonication had slightly improved tackiness values (*p* < 0.05). Manzoor et al. [17] reported that increased gelator concentration significantly improved the textural properties of the oleogels. A similar finding was also reported by Meng et al. [18]. Park et al. [19] found that oleogel’s textural properties significantly improved when the gelator concentration was more than 5%. Sharifi et al. [20] reported that applying ultrasonication to the olive oil-based oleogel preparation significantly increased the hardness of the oleogel. Application of ultrasonication on the oleogel emulsion could alter the functional properties of the fats by modifying the oil’s crystallization behavior by decreasing crystal size and producing an oleogel network that is stronger and more elastic and, consequently, obtaining the improved material textural behaviors [21]. 

### 2.2. Thermal Properties

**Figure 3:**
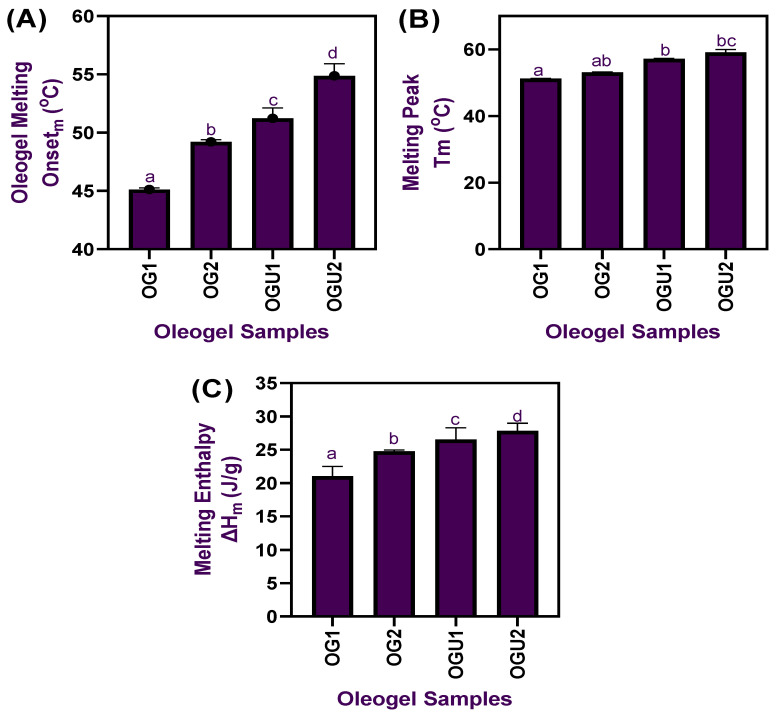
Melting onset (**A**), melting peak temperature (**B**), and melting enthalpy (**C**) of palm oil-based oleogels prepared with different concentrations of carnauba wax and the ultrasonication process.

The oleogel samples that were prepared using palm oil and different concentrations of CW and followed by an ultrasonication process were tested for various thermal properties using the DSC, and the results are shown in Figure 3. Generally, the thermal properties of oleogels are considered to be an essential tool to identify the use of plant-based fats for various applications, and they are not key parameters for the characterization of oleogels [22]. The melting onset and peak temperature of oleogels significantly differed from each other; the inclusion of CW at different concentrations significantly improved the melting temperatures of oleogels (OG1-OG2) (*p* < 0.05). Additionally, the application of ultrasonication in the oleogel (OGU1-OGU2) process and increased concentration of CW had further increased the melting temperature in the oleogels (*p* < 0.05). The OG1 onset melting temperature was recorded at 45.12 °C, and the melting peak temperature was recorded at 51.23 °C; for OG2, it was 49.21 °C for the onset temperature and 53.14 °C for the melting peak temperature. On the other hand, the ultrasonicated oleogels had slightly higher onset and peak temperatures, particularly for OGU1; the onset melting temperature was 51.23 °C. The peak temperature was recorded at 57.17 °C; for OGU2, the onset temperature was recorded at 54.87 °C, and the peak melting temperature was recorded at 59.12 °C. Overall, the results indicated that increased CW and the ultrasonication process significantly (*p* < 0.05) increased the melting temperature in the oleogels, creating versatility of these palm oil-based oleogels for various applications. Noonim et al. [10] found that applying palm oil-based oleogels as an alternative to a conventional frying medium in cooking instant noodles and the oleogels significantly improved the product quality. Furthermore, their study also found that handling the oleogels at various temperatures had not damaged the structural integrity of the oleogels. The uniqueness of the CW-based oleogels is that the CW, a thermos-reversible plant wax, has a multiversatility usage and is perfectly suitable for organogelation and a simultaneous emulsification process [23]. Furthermore, the melting enthalpy of the oleogels significantly differed (*p* < 0.05), and, overall, the enthalpy of oleogels ranged between 21.04–27.84 ΔHm (J/g). Oleogels without the ultrasonication process had a slightly low enthalpy (21.04–24.78 ΔHm (J/g)) value as compared with the ultrasonicated oleogels (26.54–27.84 ΔHm (J/g)). Co ED and Marangoni [24] reported that increased concentration of plant wax in the oleogel emulsions greatly increased the melting temperatures. The present study is in accordance with their study.

### 2.3. FTIR Spectra and X-ray Diffractions

**Figure 4:**
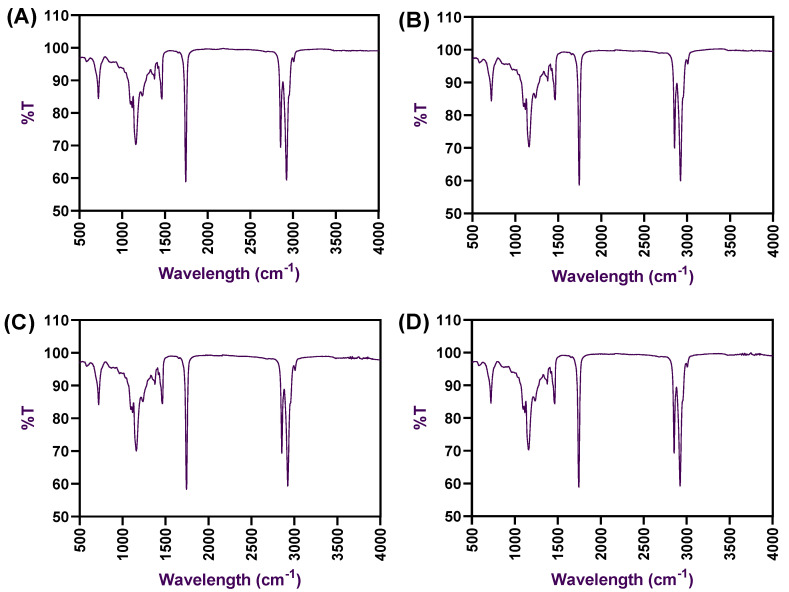
FTIR spectrum of palm oil-based oleogels (OG1 (**A**), OG2 (**B**), OGU1 (**C**), and OGU2 (**D**)) prepared with different concentrations of carnauba wax and the ultrasonication process.

**Figure 5:**
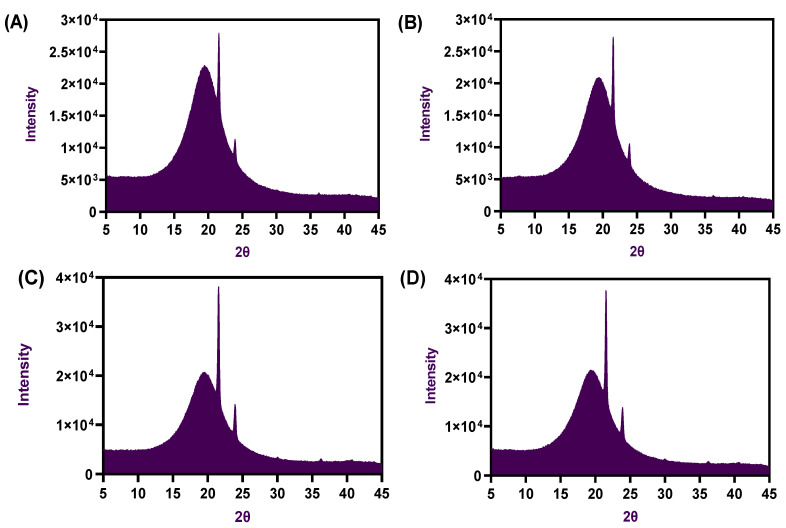
XRD patterns of palm oil-based oleogels (OG1 (**A**), OG2 (**B**), OGU1 (**C**), and OGU2 (**D**)) prepared with different concentrations of carnauba wax and the ultrasonication process.

The FTIR spectra of the oleogel samples made of palm oil with CW and processed under ultrasonication were tested, and the results are shown in Figure 4. Normally, the FTIR is considered to be a powerful technique to identify the formation of hydrogen bonding between food molecules [25]. Overall, the spectra result of oleogels exhibited a similar trend across all the samples tested. The oleogel samples showed specific peaks at different areas, particularly at around 730, 1150, 1530, 1700, 2900, and 2950 cm^−1^, respectively. Some deformation peaks were observed at around 3600 to 4000 cm^−1^, which could be due to intermolecular hydrogen bonding by exchanging protons between the alcohol, amine, amide, and/or carboxylic groups [26]. The peaks at this wavelength region are also specific to polyunsaturated fatty acids and normally less visible in oleogels. This is in accordance with the study of Thakur et al. [8]. During the FTIR spectra observation, the oleogel exhibited various peaks with medium (730–1150 cm^−1^) and vigorous intensities (1530–1700 cm^−1^ and 2900–2950 cm^−1^), which represent the intermolecular and intramolecular hydrogen bonds, respectively. This finding suggests the presence of C-H and C=O stretching in the oleogels. The peak observed between 715 and 730 cm^−1^ could be the cause of the cycloalkane bending of alkyl groups in the oleogel compositions. Peaks observed at 1160 cm^−1^ represented the C-O, C-O-O, and C-O-H stretching in the oleogels. Peaks between 1500–1600 cm^−1^ could be the effect of stretching of C-H in the methylene and methyl groups, which are the fatty acid’s backbone. Peaks found between 1600 and 1700 cm^−1^ could be the effect of stretching and vibration of the carbonyl group of esters and alkene groups and could also be due to the interesterification process by the oleogels [16]. Studies have reported that oleogel made of carnauba wax produces strong intermolecular hydrogen bonding between the oil and the wax [15,22]. Therefore, the results of FTIR spectroscopy indicated that there were no significant chemical interactions that happened by depicting similar peak positions amongst the different oleogel samples, and, mainly, the oleogel may have formed based on molecular self-assembly or building blocks, which are usually stabilized by noncovalent interactions such as hydrogen bonding, short-range Van der Waals attractive interactions (dispersion forces), and π–π stacking [17]. Several studies suggest that the formation of noncovalent interaction in the oleogels would form a semicrystalline structure [27,28]. According to the previous literature, the semicrystalline structure formation of oleogels is mainly due to the intramolecular or intermolecular hydrogen bonding during the preparation [18]. X-ray diffraction of the oleogel samples was tested, and the results are shown in Figure 5. Generally, the X-ray diffraction method is used to understand the internal structure of the oleogels [29]. The present study revealed that all the tested oleogels had formed a relatively orderly structure by CW, binding tightly with liquid palm oil without the outburst of the oil upon handling. XRD patterns of the oleogels in this study were very similar, and there were no obvious differences among the oleogels on the XRD patterns despite different wax and ultrasonication processes applied. Two common XRD peaks were observed at the wide-angle region in all oleogel samples, particularly around 21.8 and 23.9, indicating the extension of enlargement of the molecular chain in the emulsion medium and leading to the transition confirmation. The application of ultrasonication in the oleogel preparation increased the confirmation rate. Additionally, peaks at that particular region indicate the orthorhombic perpendicular subcell packing-type crystals, and this type is very similar to the crystals of margarine, spreads with a smooth texture, and has better mouthfeel properties. This is in accordance with the study of Ögütcü et al. [22]. The present study results indicated that the formation of crystals within the oleogel network was well-developed and depicted clear diffraction intensity. This is in accordance with Ghose et al. [30]. 

### 2.4. Microscopical Observations

**Figure 6:**
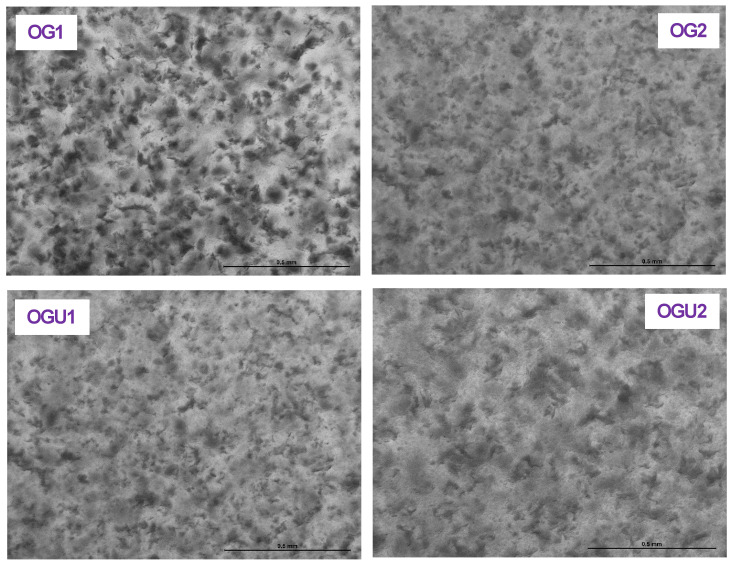
Microscopic observation of palm oil-based oleogels prepared with different concentrations of carnauba wax and the ultrasonication process.

The morphological structures of palm oil-based oleogels made of different concentrations of CW and the ultrasonication process were tested by light microscope and presented in Figure 6. Generally, the microscopical analysis of oleogels displays the differences in the sample’s microstructure due to their dominance of ingredients [16]. The results exhibited a smooth, smear-like structure of CW crystals that were smoothly dispersed in the continuous phase of liquid palm oil in all the tested oleogels. On the other hand, the level of smears in the oleogels was heavily affected by the level of CW and ultrasonication applied. Overall, this study showed that when oleogels were made of CW alone, they were congested, and a large number of smear-like structures were found all over the observed areas. Meng et al. [18], in a study, found that the addition of more concentrations of gelators in the oleogel emulsion could result in a smaller crystalline network. This is in accordance with the present study; OG2 oleogel, which contains 10% CW, showed a tiny crystalline structure compared to OG1 (5% CW). This is in agreement with the study of Yang et al. [31], which found that adding a higher concentration of gelators in the oleogel emulsion had the ability to self-assemble in the oil phase and produce a tiny and tighter crystalline network with smoother morphology. Furthermore, the oleogels made of similar CW concentrations, but processed under ultrasonication, significantly reduced the large smears and exhibited smoother and firmer crystalline networks in the oleogels. Ögütcü et al. [22] reported that droplet size of the oleogel emulsion plays a crucial role in retaining structural stability. Applying the ultrasonication process could reduce the droplet size in the oleogel emulsion and, thus, make smooth and strong structural oleogels (OGU1-OGU2). Pucas et al. [16] reported that oleogel-based spreads can have more dense structures. Szymanska et al. [32] reported that oleogels’ morphological observations are critically influenced by their compositions, particularly crystalline sizes, which are categorized as small, medium, and maximum. Small, crystalline-like oleogels normally exhibited dense and firm networks. Yu et al. [33] found that the application of ultrasonication in the oleogel preparation led to forming a small crystalline network in the oleogels. 

### 2.5. Oil Loss and Oxidative Stability

**Figure 7:**
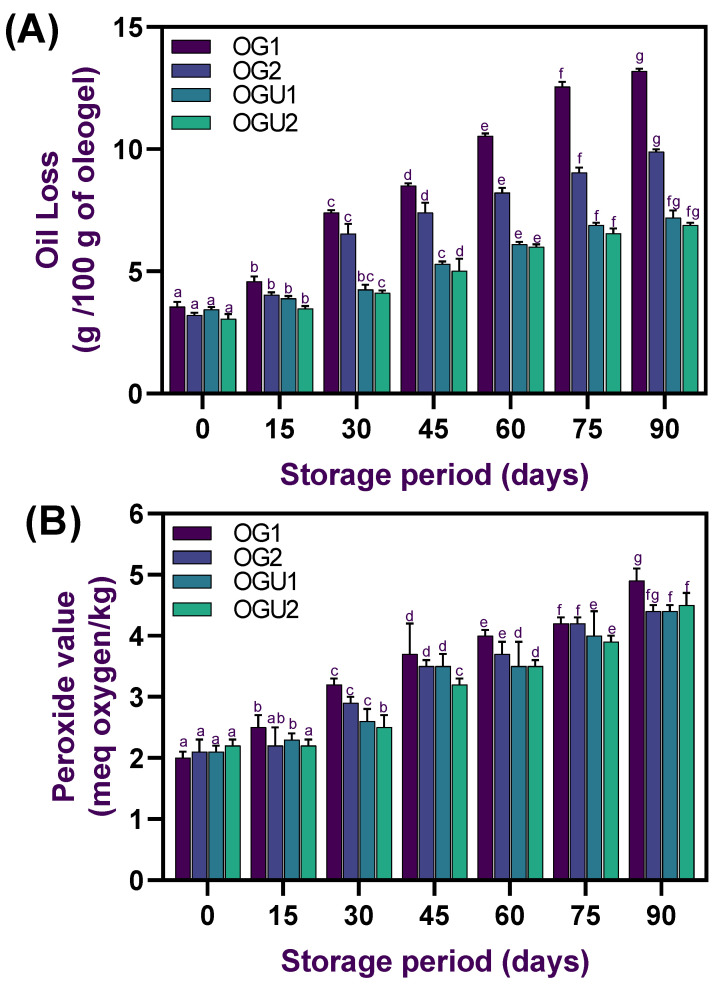
Oil loss (**A**) and peroxide value (**B**) of palm oil-based oleogels prepared with different concentrations of carnauba wax and the ultrasonication process.

The oil loss in the palm oil-based oleogel samples that were prepared with different concentrations of CW and the ultrasonication process are shown in Figure 7. The oil-binding capacity of wax oleogels is one of the main characteristics that define their functionality in food products. Generally, the fresh oleogels show no oil loss; however, the severity of loss exhibits only when the oleogels are stored for a prolonged period. This study tested the oil loss in the oleogels every 15 days for a period of 90 days, and the results showed that an increased storage period significantly increased the oil loss in the oleogels (*p* < 0.05). On earlier days of the storage, the oil loss was very rapid and slowly remained stable; however, throughout the storage period, a loss of oil was continuously recorded in all the samples. Furthermore, the results showed that different concentrations of CW and the ultrasonication process significantly affected the oil loss in the oleogels (*p* < 0.05). A low concentration of CW poorly withstood the oil in the oleogel structure as compared with the higher CW concentration (*p* < 0.05). On the other hand, the ultrasonication process increased the structural stability of the oleogels and improved the strong bonding between oil and the wax samples, which resulted in a lower oil loss (*p* < 0.05). Meng et al. [18] reported that increased gelator concentration significantly decreased the oil loss in the oleogels. Bascuas et al. [34] also observed similar findings when edible oleogels stored for a prolonged period experienced severe oil loss, despite the different oil composition and gelator concentration used. Gaudino et al. [35] reported that waxes could be able to provide a crystalline structure that provides tolerances against processing conditions involving mixing and shearing while not falling apart and releasing all the bound oil. Blake et al. [36] reported that the oil loss in the oleogels during minimal to moderate processing conditions is mainly attributed to three important properties, which include structural properties, wax crystal size, and spatial distribution and displacements. In another study, Meng et al. [18] reported that semicrystallization in the oleogel polymer network could induce oil loss in the oleogels. The oxidative stability of the oleogels samples was examined by measuring peroxide value during storage under ambient temperature for 90 days. The obtained results are shown in Figure 7. Overall, a steady increase in PV values was observed in all oleogel samples throughout the storage period (*p* < 0.05). Among the samples, oleogels made of CW at 5% concentration exhibited a significantly higher level of PV than the others (*p* < 0.05). On the other hand, the ultrasonication-processed oleogel samples with different concentrations of CW did not significantly differ (*p* > 0.05); however, their oxidative stability was slightly higher than the other tested samples (*p* < 0.05). These results indicate that oleogels made of CW with a higher concentration and with or without the ultrasonication process could be able to control the peroxide values. According to Codex Alimentarius [37], if oil-based food products contain a peroxide value of less than 15–20 meg kg^−1^, it is considered safe. Peroxidation in oil-based products is generally produced upon prolonged storage and could be the effect of autoxidation. Unsaturated fatty acids are the key sources of peroxidation, and, particularly, polyunsaturated fatty acids are susceptible in comparison with monounsaturated fatty acids. In this study, the free mono- and polyunsaturated fatty acids could strongly integrate with the oleogels by the increased CW concentration and ultrasonication process and thus slightly lowered the level of peroxidation in the oleogels. Valoppi et al. [38] reported that ultrasonication treatment on the oleogel emulsion could induce physical barriers against various external stimuli that promote degradations. 

## 3. Conclusions

Using oleogels instead of chemically modified trans fat has proven to be successful without negatively impacting food quality. Moreover, a growing number of consumers rely on it for its health benefits. There are numerous ways to produce oleogels; however, their stability is still a major concern. The present study evaluated ultrasonication in comparison with a homogenizer to improve the properties and storage stability of palm oil-based oleogels prepared using carnauba wax at different concentrations (5% or 10%). The results showed that ultrasonication improved yellowness and enhanced the textural properties, including hardness, stickiness, and tackiness of oleogels. Moreover, thermal properties such as melting enthalpy, melting onset, and peak temperature were found to be higher in oleogels processed with ultrasonication. FTIR spectra revealed that ultrasonication promotes the formation of more intermolecular hydrogen bonds in oleogels. In addition, XRD indicated that ultrasonication-processed oleogels had more crystalline polymorphs. The microscopic observation found that oleogels processed with ultrasonication at 10% carnauba wax had a smooth and strong crystalline structure. Furthermore, it showed lower oil loss and higher peroxidation stability during storage of 90 days under ambient conditions. Overall, this study recommends that use of ultrasonication could improve the properties and enhance storage stability of palm oil-based oleogels, which could be used as a functional food. 

## 4. Materials and Methods

### 4.1. Raw material, Chemicals, and Reagents

The refined palm oil at a commercial grade was purchased from the local supermarket at Surat Thani province, Thailand. The phyto-gelators such as carnauba wax flakes were purchased from DCMC corporation Co., Ltd., Bangkok, Thailand. All the chemicals and reagents used in this study for various analyses were purchased from Sigma (St. Louis, MO, USA).

### 4.2. Oleogel Preparation

**Figure 8:**
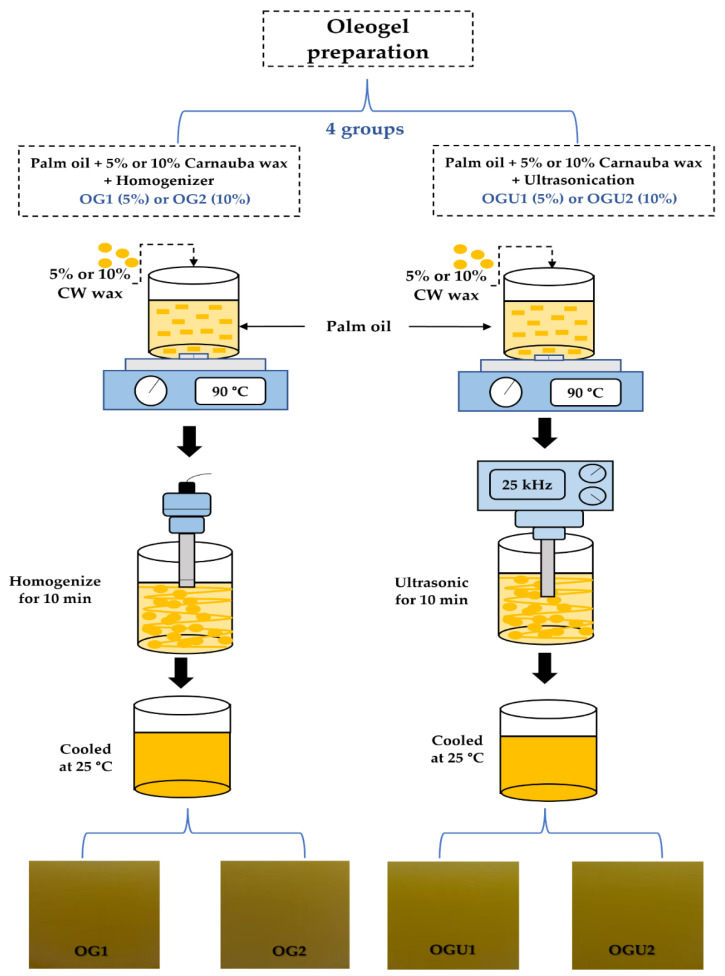
Infographic presentation of palm oil-based oleogel prepared with different CW concentrations and the ultrasonication process.

The oleogel used in this study was prepared in accordance with the method of Noonim et al. [10], with slight modifications. Four groups of oleogels (OG1 (palm oil + 5% carnauba wax), OG2 (palm oil + 10% carnauba wax), OGU1 (palm oil + 5% carnauba wax + ultrasonicated), and OGU2 (palm oil + 10% + ultrasonicated)) were prepared and tested in this study. For OG1 and OG2 preparation, the carnauba wax at a selected concentration was mixed well in the palm oil, using a temperature-controlled hot plate which was set to 90 °C, once the carnauba wax was completely dissolved in the palm oil; then, the mixture was removed from the hot plate and vigorously homogenized with the handheld homogenizer for 10 min, and after that, the oleogel mixture was cooled at ambient temperature. For OGU oleogels, all the steps for preparing oleogels were similar to the process shown above for the OG; however, the homogenization process with handheld homogenizer was replaced by the ultrasonication process and was performed by using a portable ultrasonic processor (Hielscher UP200Ht, Hielscher Ultrasound Technology, Germany) that was equipped with a probe tip (40 mm) and processed at a constant 25 kHz for 10 min, followed by cooling at ambient temperature. Figure 8 shows the infographic of the oleogel preparations.

### 4.3. Analysis

#### 4.3.1. Color Characteristics

Color characteristics, including lightness (L*), redness (a*), and yellowness (b*), were recorded on the oleogels at random points by using the Hunter LAB colorimeter (Hunter Associates Laboratory, Inc. Reston, VA, USA). The total color characteristics of the oleogels were calculated using the L*, a*, and b* values by following the equation proposed by Tiga et al. [39].

$$∆E^{*}=\sqrt{∆L^{*2}+∆a^{*2}+∆b^{*2}}$$

#### 4.3.2. Texture Profile

Texture profile (hardness, stickiness, and tackiness) in the oleogels was measured in accordance with the method of Gravelle et al. [40]. Prior to analysis, the oleogels were place in a 100 mL beaker at room temperature, and then, the textural analyzer (LFRA 4500, Brookfield Engineering, Middleborough, England) probe (40 mm diameter flat acrylic probe) was penetrated around 15 mm into the oleogels from the surface at a speed of 1.0 mm/s. The obtained data from the texture analyzer were calculated, and the results were presented in force (N).

#### 4.3.3. Thermal Properties

The thermal analysis of oleogels, including melting onset temperature, melting peak temperature, and melting process enthalpy, was measured using a differential scanning calorimetry (DSC, Perkin Elmer 4000 series, Groningen, The Netherlands) equipped with Pryis 1 Manager Software by following the method of Ogutcu and Yilmaz [22]. Due to the fact that the cooling might promote the crystallization process, in this study, only the melting (heating) experiment was performed in the oleogel samples.

#### 4.3.4. Morphological Observations

A polarized light microscope (PLM) (Olympus BX51, Olympus Co., Ltd., Tokyo, Japan) equipped with a CCD color digital camera (Canon, Japan) was used to observe the crystalline structures of the oleogel emulsions. Small amounts of fresh oleogel sample were placed on glass microscope slides, and these slides were covered with glass coverslips, and the PLM was used to observe the morphological observation. In order to observe the polymorphic forms of the crystals of oleogels, fully polarized digital images were taken at 50x zoom level at room temperature.

#### 4.3.5. XRD Analysis

XRD patterns of the oleogels were tested based on the method of Sahu et al. [41] by using an X-ray diffractometer (PA Analytical, Eindhoven, The Netherlands). Angular scans in the oleogels were performed from 5–50° at 0.026°/min scan rate with a copper source X-ray tube, α = 1.54 Å. X-ray generator power was set to 40 K V and 30 mA.

#### 4.3.6. FTIR Spectroscopy

Oleogels were measured by FTIR spectroscopy by using the method of Totosaus et al. [42]. Prior to measurement, an oleogel film was prepared by placing the sample between two glass microscope slides and compressing the film until it reached a thin thickness. After that, single-beam spectra of the oleogel samples were collected against the background of air and presented in a transmission unit using an FTIR spectrophotometer (Thermo Electron Corp., Madison, WI, USA). The samples were measured for FTIR in the wavelength range between 4500 and 500 cm^−1^ with a nominal resolution of 10 cm^−1^. For spectral analysis, OPUS 3.0 data collection software (Bruker CO., Ltd. Billerica, MA, USA) was used.

#### 4.3.7. Oil loss and Oxidative Stability of Oleogels

Oleogels were stored in transparent containers for 90 days at room temperature, and after every 15 days, samples were removed from the containers and tested for oil loss and oxidative stability. Prior to oxidative stability analysis, all the oleogel samples were reheated to 45 °C to transform from solid to liquid, and after that, they were used for all the following analyses.

##### Oil Loss

Oil loss in the oleogel was determined by following the method of Doan et al. [43], with slight modifications. Oil loss in the oleogels were measured at an interval of every 15 days for a total period of 90 days. In this experiment, a funnel with filter paper was placed above an Erlenmeyer flask, into which the dripping liquid oil from the oleogels was collected. Prior to loading the oleogel, the weight of the funnel, the filter paper, and the Erlenmeyer flask were weighed and referred to as M1. Afterward, 10 g of oleogel (M3) were weighed and placed in the funnel. At each interval, samples were collected using a flat, small spatula. Once again, the funnel, the filter paper, and the flask containing the liquid oil was weighed (M2). The results were calculated using the following formula, and oil loss in the oleogels were reported as g oil loss per 100 g oleogel.

$$\mathrm{Oil}\;\mathrm{loss}\;(g\;\mathrm{oil}\;\mathrm{loss}\;\mathrm{per}\;100\;g\;\mathrm{oleogel})=\frac{\left[M2-M1\;\right]}{M3}\times \;100\%$$

##### Peroxide Value (PV)

PV value in the oleogel was measured using the titration method in accordance with the method of Pudtikajorn and Benjakul [44]. A total of 0.1 g of oleogel samples was mixed with 25 mL of acetic acid (3)/chloroform (2) solution, and then, 1 mL of saturated potassium iodide was added to the samples, followed by the addition of 75 mL distilled water. Then, the reaction mixture was thoroughly mixed and kept in a dark place for 5 min. Afterwards, the mixture was titrated with 0.01 N of sodium thiosulfate after starch solution (1%) was added as an indicator, and the titration endpoint was obtained when the dark blue of the sample solution faded to pink. The following equation was used to determine the PV value.

PV (meq oxygen per kg)=[V X M X 1000 ]/W

where V is the volume of sodium thiosulfate (mL); M is the concentration of sodium thiosulfate (N); and W is the weight of the sample (g).

### 4.4. Statistical Analysis

All analyses were conducted in triplicate in this study, and all data are expressed as mean ± standard deviation. For significance testing, a one-way analysis of variance (ANOVA) and Duncan’s multiple ranges post hoc test were conducted using *p* < 0.05 as the standard level of significance. Statistical analyses were carried out using the Statistical Package for the Social Sciences (SPSS) from SPSS Inc, Chicago, IL, USA.

## Data Availability

This article is already contained all the available data.
